# Supplementary material for: Nonnegative/Binary matrix factorization with a D-Wave quantum annealer
Source: PLoS One. 2018 Dec 10;13(12):e0206653. doi: 10.1371/journal.pone.0206653 (PMC6287781; doi:10.1371/journal.pone.0206653)
Supplement: S1 Appendix — (PDF) [file pone.0206653.s001.pdf]

S1 Appendix.

Here we show how repeatedly solving Eq. 3 is equivalent to solving one instance of Eq. 2. The relationship between these two equations can be seen by examining the relevant quantities from an elementwise perspective,

$$||V - WX||_F^2 = \sum_{i=1}^{m} \sum_{j=1}^{n} \left(V_{ji} - \sum_{l=1}^{k} W_{jl} X_{li} \right)^2 = \sum_{i=1}^{m} ||V_i - WX_i||_2^2 \qquad (1)$$

Note that minimizing each term in the sum on the right hand side of Eq. 1 as a function of X_i amounts to solving an instance of Eq. 3 – the $\mathbf{q}$ in Eq. 3 is equivalent to X_i in Eq. 1. Each term in the sum on the right hand side of Eq. 1 depends only on one column of X, and each column of X appears in only one term. Therefore, when these terms are simultaneously minimized, the left hand side of Eq. 1 will be minimized as a function of X. Since minimizing the left hand side of Eq. 1 as a function of X is equivalent to solving Eq. 2, we can conclude that repeatedly solving Eq. 3 is equivalent to solving Eq. 2.
